# Gender Differences in Attitudes Toward Aging and Its Longitudinal Impact on Psychological Health

**DOI:** 10.1093/geroni/igaa057.1040

**Published:** 2020-12-16

**Authors:** Eun Young Choi, Yujin Franco, Elizabeth Zelinski

**Affiliations:** 1 Leonard Davis School of Gerontology, Los Angeles, California, United States; 2 University of Southern California, Los Angeles, California, United States

## Abstract

Individuals with negative attitudes towards own aging (ATOA) experience worse psychological health in later life. At the intersection of sexism and ageism, women are likely to have greater concerns about growing older and hold more negative views of aging than their men counterparts. However, the impact of gender on the relationship between ATOA and psychological health is unclear. Moving forward, the current study aims to examine 1) gender differences in longitudinal changes in ATOA and 2) whether gender moderates the association of ATOA with cognitive function and depressive symptoms. Using three waves (2008, 2012, and 2016) from the Health and Retirement Study, a total of 6,675 adults aged 50+ (60% female) were analyzed. A series of multilevel growth curve analyses were performed to investigate the 8-year changes in ATOA and within- and between-person effects of ATOA on cognitive function and depressive symptoms. The models controlled for demographic, socio-economic, and physical health characteristics. Women had more negative ATOA at baseline compared to men, but not in rates of change. When levels of ATOA were more negative, both cognitive performance and depressive symptoms were poorer over time between individuals as well as within-person. We found that the detrimental effects of negative ATOA on depressive symptoms were stronger for women, but there were no significant gender differences in relation to cognitive functioning. Our findings demonstrated that women view aging more unfavorably than men, and the effects of endorsing negative ATOA are more pronounced on women’s mental health.

